# A double‐hit High‐grade B‐cell lymphoma with three‐way translocation t(3;8;14)(q27;q24;q32) involving BCL6, MYC, and IGH

**DOI:** 10.1002/ccr3.1871

**Published:** 2018-10-26

**Authors:** Elena De Paoli, Laura Bandiera, Emanuele Ravano, Clara Cesana, Giovanni Grillo, Valentina Mancini, Gabriella De Canal, Emanuela Bonoldi, Silvia Soriani

**Affiliations:** ^1^ Department of Laboratory Medicine ASST Grande Ospedale Metropolitano Niguarda Milan Italy; ^2^ Division of Hematology ASST Grande Ospedale Metropolitano Niguarda Milan Italy

**Keywords:** BCL6, double‐hit lymphoma, High‐grade B‐cell lymphomas, MYC, t(3;8;14), three‐way translocation

## Abstract

We describe an High‐grade B‐cell lymphoma case, in which a complex translocation t(3;8;14) with effects on the genes BCL6, MYC, and IGH, was detected. This case could be the first double‐hit lymphoma with a single chromosome rearrangement causing the double effect with three genes involved.

## 1 INTRODUCTION

The World Health Organization (WHO) updated Classification of tumors of Haematopoietic and Lymphoid Tissues (2016) introduced a new subset of mature B‐cell neoplasms, High‐grade B‐cell lymphoma (HGBL) with MYC and BCL2 and/or BCL6 rearrangements.

This new subtype is now commonly referred to as double‐hit lymphoma (DHL) or Triple‐Hit Lymphoma (THL).^1^ DHL and THL are associated with an aggressive clinical course and resistance to conventional chemotherapy regimens. The main genetic feature is the occurrence of a translocation involving *MYC* (8q24) together with rearrangements involving *BCL2* (18q21) and/or *BCL6* (3q27) genes. The involvement of *BCL6* is less frequent compared to *BCL2* (10% and 73%, respectively).^2^ It has been hypothesized that this different rate can be due to either *MYC* ^+^/*BCL6* ^+^ DHL being less frequent or because of an underestimation of *BCL6* rearrangements as recorded in the Mitelman's database.^3^ Indeed *BCL6* breakpoints are located in the subtelomeric region of the long arm of chromosome 3 and may be missed by standard chromosomes analysis.^4^ Another hallmark of DHL is the greater degree of cytogenetic complexity compared to Burkitt lymphoma (BL) with which it often shares morphologic features. In particular, *MYC* ^+^/*BCL2* ^+^ DHL and THL are cytogenetically as complex as DLBCL, instead of *MYC* ^+^/*BCL6* ^+^ DHL that can show a simple karyotype in half cases.^5^ *MYC* ^+^/*BCL6* ^+^ DHL shares many morphological features with *MYC* ^+^/*BCL2* ^+^ DHL, but immunohistochemistry reveals some differences because *MYC* ^+^/*BCL6* ^+^ DHL is mostly BCL2 negative, with decreased CD10 expression but apparently increased IRF4/MUM‐1 immunoreaction.^5^ Furthermore, it is reported that *MYC* ^+^/*BCL6* ^+^ DHL is aggressive, with a recurrent extra‐nodal localization, poor prognosis, and a germinal center (GC) type phenotype.^5^, ^6^

Here, we report a patient presenting with clinical conditions suggesting acute leukemia, then concluded as a case of DHL associated with a three‐way chromosomal translocation t(3;8;14)(q27;q24;q32) involving *BCL6*,* MYC,* and *IGH* genes, respectively, confirmed by fluorescence in situ hybridization (FISH) performed on metaphase cells.

## 2 MATERIALS AND METHODS

### 2.1 Flow cytometry, cytogenetic analysis, and FISH

Peripheral blood (PB) and bone marrow (BM) aspirate smear, cytometric examinations, and BM biopsy were performed and processed according to standard procedures.

Unstimulated and DSP30+IL2‐stimulated BM cultures were obtained for chromosome and FISH analysis (Chang Medium BMC, Irvine Scientific, by TechnoGenetics, Milan, Italy; DSP30, Tib MolBiol srl,Genova, Italy; IL‐2, Gibco by Life Technologies, Italy).^7^ Harvesting and slide preparation were performed according to standard cytogenetic protocols, and chromosome analysis was performed by QFQ‐banding technique. At least 20 metaphases from both cultures were analyzed. FISH analysis was carried out by commercially available probes, according to the manufacturer's instructions: MYC Breakapart Probe and BCL6 Breakapart Probe (catalog number: LPH 035) (Cytocell Ltd., Cambridge, UK), XL IGH plus Break Apart Probe (MetaSystems, Althlussheim, Germany), LSI 1p36/1q25 and LSI 19q13/19p13 Dual Color Probe and D13S319/13q34 FISH Probe Kit (Vysis/Abbott, IL, USA). At FISH,available metaphases and at least 100 not‐overlapping interphase nuclei were evaluated. Karyotypes and FISH results were described according to International System for Human Cytogenetic Nomenclature (ISCN 2016).^8^

## 3 CASE REPORT

### 3.1 Clinical presentation

A 66‐year‐old woman was admitted to our hospital due to dizziness, hearing loss, facial hypoesthesia, and muco‐cutaneous bleeding. A physical examination revealed bruises, hematomas, and petecchiae all over the body and in the mouth. A complete neurological examination highlighted left periocular and perioral hypoesthesia, postural instability and left hearing loss. A brain computed tomography (CT) scan was performed, showing a left cerebral subdural bleeding without mass effect. A pathological meningeal contrast enhancement in the left fronto‐temporal hemisphere was demonstrated by magnetic resonance imaging (MRI), together with an infiltrative mass of cochlea, semicircular canals, and vestibulocochlear nerve, compatible with disease localization. Positron emission tomography and a CT scan of neck/chest/abdomen were negative except for homogeneous splenomegaly (15 cm longitudinal diameter). Informed consent about authorization for instrumental examinations and genetic analysis on biological sample, processing of personal data, and sample storage was obtained. All clinical findings together with BM morphologic, immunophenotypical, cytogenetic, and FISH evaluation led to a diagnosis of DHL with central nervous system and BM involvement, clinical stage IV.

The patient received a first cycle of R‐HyperCVAD B (high doses of Methotrexate and Cytarabine). The chemotherapy was well tolerated with resolution of all the symptoms. However, during hospitalization a clinical relapse arose, characterized by headache, confusion, disorientation and recurrence of dizziness, ipoesthesia, and hearing loss was also observed. A brain MRI confirmed the progression of the disease. After the sixth cycle of R‐ICE (Rituximab – Ifosfamide – Carboplatin AUC 5‐ Etoposide) complete remission (CR) was recorded by brain MRI and BM morphologic, immunophenotypical, cytogenetic, and FISH evaluation. An autologous PB stem cell transplantation was subsequently and successfully performed. The patient is now alive and in CR

## 4 RESULTS

**Figure 1:**
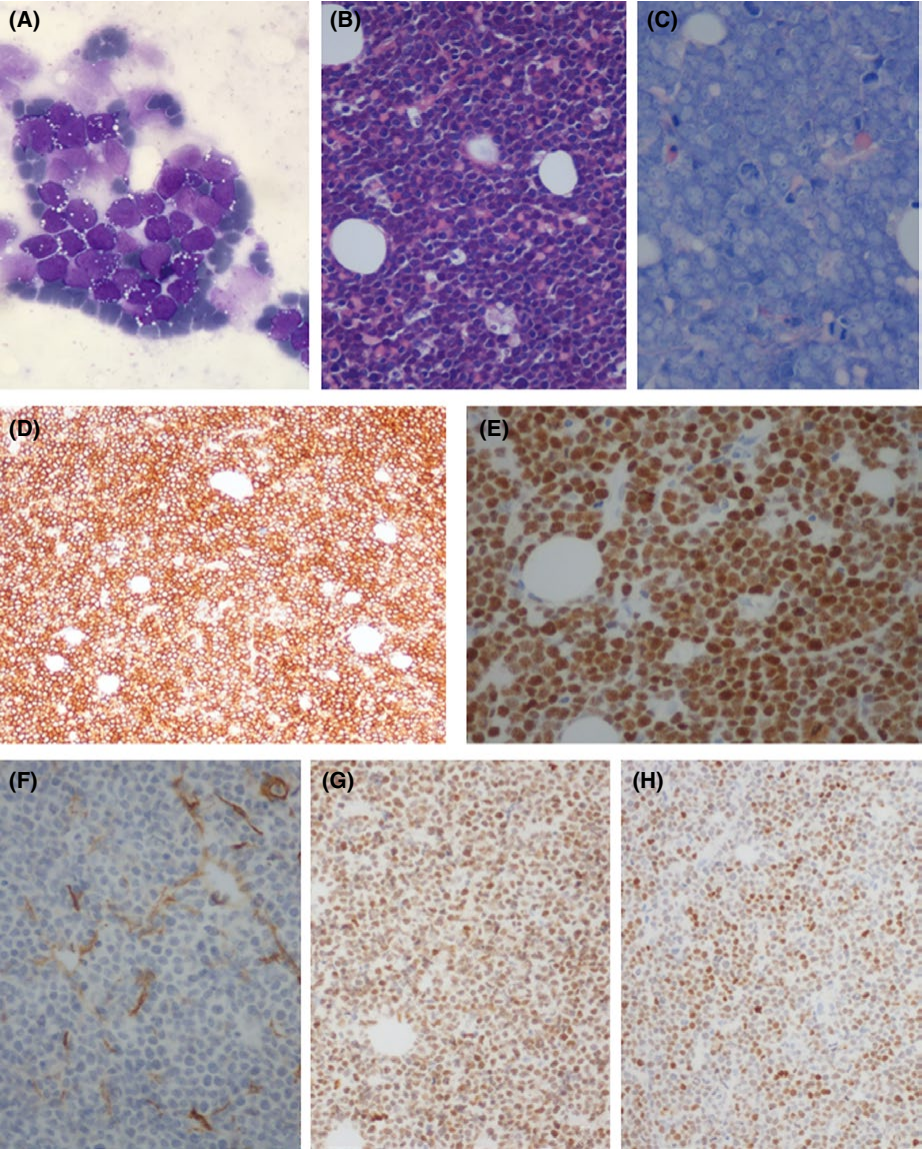
BM morphological evaluation: aspirate smear (1000x) (A), hematoxylin and eosin (200x) (B), Giemsa (400x) (C); Immunohistochemistry performed on BM: CD20 (100x) (D), MYC (200x) (E), COO‐panel with CD10 (100x) (F), BCL6 (100x) (G), and IRF4 (100x) (H)

Peripheral blood examination showed WBC 18.0 × 10^9^/L, HGB 13.5 g/dL, PLT 14 × 10^9^/L, LDH 3400 U/L, with no significant abnormalities in renal and hepatic functionality or in coagulation parameters. A population of atypical lymphocytes with intense basophilic cytoplasm, high nucleo/cytoplasmatic ratio, and frequent vacuoli, was observed at morphological evaluation, representing 17% of cells (Figure 1A).

BM aspirate smear showed large‐size lymphoid B cells, immunophenotypically CD45^+^ CD19^+^ CD20^+^ cytCD22^+^ CD38^+^ CD45RA^+^ cytCD79a^+^ HLADR^+^ sIgl^+/−^ CD1a^−^ CD2^−^ cytCD3^−^ CD4^−^ CD5^−^ CD7^−^ CD8^−^ CD10^−^ CD11b^−^ CD11c^−^ CD13^−^ CD14^−^ CD15^−^ CD16^−^ CD33^−^ CD34^−^ cytCD41^−^ CD56^−^ cytCD61^−^ CD64^−^ CD117^−^ CD235a^−^ cytMPO^−^.

Bone marrow biopsy showed hypercellularity (90%) with complete replacement by diffuse, massive infiltrate (>90% of cell population), consisting of medium/large size monotonous lymphocytic cells with blastic appearance, scant cytoplasm, inconspicuous nucleoli, and many mitotic Figures (Figure 1B‐C), expressing B markers CD20^+^, BCL6^+^, MUM1^+^, BCL2^+^, MYC^+^ (>90%), CD10^−^, CD30^−^ («1%), CD5^−^, TdT^−^, CD34^−^ (Figure 1D‐H). Cytogenetic study was needed.

**Figure 2:**
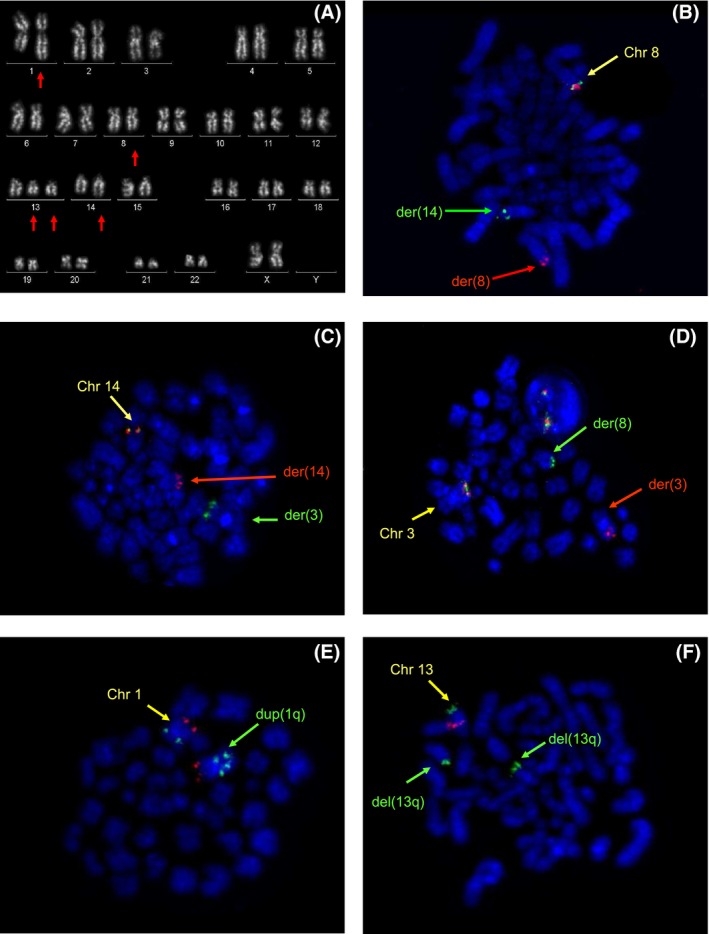
QFQ‐banding on chromosomes obtained from BM cultures revealed the following complex karyotype (A). Arrows indicate rearranged chromosomes. FISH analysis performed on metaphases using MYC (B), IGH (C), and BCL6 (D) Break Apart probes revealed the presence of a three‐way chromosome rearrangement. Additional chromosome anomalies were investigated using specific probes: MEGF6 and TP73 in 1p36 (red) and ABL2 in 1q25 (green) (E); locus D13S319 in 13q14 (red) and 13q34 region (green) (F)

Subsequent cytogenetic analysis showed the presence of multiple clonal aberrations in 11 over 26 analyzed metaphases. The karyotype was: 47,XX,dup(1)(q25q32),?del(8)(q24),+13,del(13)(q13q21)x2,add(14)(q3?1)[cp11]/46,XX[15] (Figure 2A).

*MYC* and *IGH* status were at first investigated using Break apart FISH probe. *MYC* rearrangement was observed in 30/100 interphase nuclei and FISH analysis on DAPI counterstained metaphases showed one yellow fusion signal (1Y) on normal chromosome 8, one red signal (1R) on derivative chromosome 8 and one green signal (1G) on derivative chromosome 14 (Figure 2B). *IGH* rearrangement was observed in 24/100 nuclei and on metaphases we found that 1G, corresponding to the *5’IGH* end, localized on the telomeric region of the long arm of a chromosome 3 (Figure 2C). So we tested the BCL6 Break Apart probe to confirm the gene's involvement: rearrangement was observed in 24/100 nuclei and on metaphases we observed 1G, corresponding to the *5’BCL6* end, localized on the derivative chromosome 8 (Figure 2D). Therefore, the two “hits” *MYC* and *BCL6* were not independent events but joined the same single mechanism itself, more exactly a translocation involving three chromosomes t(3;8;14)(q27;q24;q32). We also performed FISH probes to characterize additional chromosome anomalies observed in the karyotype: we confirmed a duplication of 1q25 including the *ABL2* gene and an interstitial deletion in 13q14, which is supposed to be duplicated at a later time (Figure 2E‐F). FISH results allowed to better define the karyotype as follows: 47,XX,dup(1)(q25q32),?del(8)(q24),+13,del(13)(q13q21)x2,add(14)(q3?1)[cp11]/46,XX[15].ish dup(1)(q25q32)(MEGF6+,ABL2++),t(3;8;14)(q27;q24;q32)(3'BCL6+,5'BCL6‐,5'IgH+;5'MYC+,3'MYC‐,5'BCL6+;3'IgH+,5'IgH‐,3'MYC+),del(13)(q13q21)(D13S319‐,LAMP1+)x2.

## 5 DISCUSSION

High‐grade B‐cell lymphoma is a group of aggressive mature B‐cell lymphomas (BCL) with intermediate features between DLBCL, NOS, and BL. Approximately 40% of all BCL are characterized by the presence of chromosome rearrangements,^4^ usually an oncogene is juxtaposed to an immunoglobulin gene (commonly *IGH*, less frequently *IGK,* or *IGL*) leading to gene overexpression. An important subset of these lymphomas is DHL or THL, bearing both a *MYC* involvement in addition to *BCL2* and/or *BCL6* rearrangements.^9^ These multiple‐hit lymphomas are associated with a very aggressive clinical course and resistance to conventional chemotherapy.^10^, ^11^, ^12^ Because of their biological, genetic and clinical features, DHL and THL are listed together as a specific category in the 2016 update of WHO classification.^1^ This revision highlights the clinical relevance of the characterization of molecular features of these disorders, therefore a specific genetic classification is always required. In this report, we describe a patient presenting seemingly as acute leukemia at first, then revealed as a DHL associated with an uncommon rearrangement, a three‐way chromosomal translocation t(3;8;14)(q27;q24;q32) involving *BCL6*,* MYC,* and *IGH* genes respectively. Focusing on patient's features, our case resembles the one recently described by Minakata et al.^13^ Both cases show a DHL with three‐way chromosome rearrangement sharing the involvement of *MYC* and *IGH* genes, but the third gene involved is *BCL2* in Minakata's case and *BCL6* in ours. Since Minakata et al had performed FISH analysis with locus‐specific probes on interphase nuclei solely, they could only speculate about the molecular rearrangement underlying the FISH results they had observed. We performed FISH both on interphase nuclei and metaphases obtained from BM cultures: this allowed detecting the presence of a translocation including chromosomes 3, 8 and 14. Finally we have the chromosome‐based FISH evidence that *MYC* and *BCL6* are involved together in a sole but three‐way chromosomal translocation. To the best of our knowledge this is the first reported *MYC* ^+^/*BCL6* ^+^ DHL case where both hits are involved in a single complex rearrangement.

Double‐hit lymphoma is well described in literature but reported cases with complex rearrangements are few, although the records are increasing.^13^, ^14^, ^15^ We believe that this lack of data is due to the method used for the analysis. Usually, *MYC*,* BCL2* and *BCL6* rearrangements are investigated by FISH performed on formalin‐fixed paraffin‐embedded lymph node tissue sections, due to disease's localization, using a locus‐specific probe. This method is very useful to investigate gene's rearrangement directly on tissues processed for histopathological diagnostics, but the analysis is therefore performed on the interphase nuclei, thus it does not allow to identify either the partner of the rearrangement or the effective underlying mechanism, as on the contrary FISH on metaphases enables. Hence we suppose that the amount of multiple‐way translocation in DHL could be underestimate because misunderstood as independent events. *MYC* ^+^/*BCL6* ^+^ DHL is aggressive in any case, whatever the molecular mechanism underlying is. It is well known that conventional chromosome analysis is often unsuitable to detect subtle rearrangements, mostly when they are part of a complex karyotype and the quality of the chromosomes is poor, as it is often observed in metaphases derived from BM cultures. Nevertheless, standard cytogenetic analysis should be carried out whenever possible, because it can disclose the presence of additional chromosome anomalies, not identified by FISH performed with locus‐specific probes, that could involve important genes and consequently change the response to therapy.

## CONFLICT OF INTEREST

None declared.

## AUTHORS' CONTRIBUTIONS

EDP: provided conception of the study, acquisition, analysis and interpretation of FISH, and cytogenetic data, drafting the article; LB, EB: supplied histological and immunohistochemical evaluation, data reporting;CC: supplied immunophenotyping, data reporting; ER, VM, GG: provided clinical support and data reporting; GDC: supplied analysis and interpretation of FISH, and cytogenetic data; SS: supplied analysis and interpretation of FISH, and cytogenetic data, drafting the article, critical supervision, and gave final approval of the version.

## References

[1] Swerdlow SH , Campo E , Lee Harris N , et al. WHO classification of tumours of haematopoietic and lymphoid tissues (Vol. 2; Revised 4th edn),2017.

[2] Haberl S , Haferlach T , Stengel A , Jeromin S , Kern W , Haferlach C . MYC rearranged B‐cell neoplasms: impact of genetics on classification. Cancer Genet. 2016;209:431‐439. 27810071 10.1016/j.cancergen.2016.08.007

[3] National Cancer Institute . Cancer Genome Anatomy Project, https://cgap.nci.nih.gov/Chromosomes/Mitelman. Accessed March 22, 2018.

[4] Aukema SM , Siebert R , Schuuring E , et al. Double‐hit B‐cell lymphomas. Blood. 2011;117(8):2319‐2331. 21119107 10.1182/blood-2010-09-297879

[5] Pillai RK , Sathanoori M , Van Oss SB , Swerdlow SH . Double‐hit B‐cell lymphomas with BCL6 and MYC translocations are aggressive, frequently extranodal lymphomas distinct from BCL2 double‐hit B‐cell lymphomas. Am J Surg Pathol. 2013;37(3):323‐332. 23348205 10.1097/PAS.0b013e31826cebad

[6] Ye Q , Xu‐Monette ZY , Tzankov A , et al. Prognostic impact of concurrent MYC and BCL6 rearrangements and expression in de novo diffuse large B‐cell lymphoma. Oncotarget. 2016;7(3):2401‐2416. 26573234 10.18632/oncotarget.6262 PMC4823044

[7] Dicker F , Schnittger S , Haferlach T , Kern W , Schoch C . Immunostimulatory oligonucleotide‐induced metaphase cytogenetics detect chromosomal aberrations in 80% of CLL patients: a study of 132 CLL cases with correlation to FISH, IgVH status, and CD38 expression. Blood. 2006;108:3152‐3160. 16840733 10.1182/blood-2006-02-005322

[8] McGowan‐Jordan J , Simons A , Schmid M . ISCN an international system for Human Cytogenomic Nomenclature. 2016.

[9] Swerdlow SH . Diagnosis of ‘double hit’ diffuse large B‐cell lymphoma and B‐cell lymphoma, unclassifiable, with features intermediate between DLBCL and Burkitt lymphoma: when and how, FISH versus IHC. Am Soc Hematol. 2014;1:90‐99. 10.1182/asheducation-2014.1.90 25696840

[10] Bacher U , Haferlach T , Alpermann T , Kern W , Schnittger S , Haferlach C . Several lymphomaspecific genetic events in parallel can be found in mature B‐cell neoplasms. Genes Chromosomes Cancer. 2011;50:43‐50. 20960563 10.1002/gcc.20831

[11] Horn H , Ziepert M , Becher C , et al. MYC status in concert with BCL2 and BCL6 expression predicts outcome in diffuse large B‐cell lymphoma. Blood. 2013;121:2253‐2263. 23335369 10.1182/blood-2012-06-435842

[12] Yamazaki T , Ohno H . Double‐hit lymphoma with t(8;14)(q24;q32) and t(12;14)(q24;q32) chromosomal translocations. Int Med. 2011;50:2659‐2662. 10.2169/internalmedicine.50.5815 22041376

[13] Minakata D , Sato K , Ikeda T , et al. A leukemic double‐hit follicular lymphoma associated with a complex variant translocation, t(8;14;18)(q24;q32;q21), involving BCL2, MYC, and IGH. Cancer Genet. 2018;220:44‐48. 29310838 10.1016/j.cancergen.2017.11.007

[14] Kato M , Miura I . Double‐hit lymphomas and complex variant translocations of t(14;18)(q32;q21.3). Ann Hematol. 2013;92(12):1723‐1725. 23604433 10.1007/s00277-013-1756-7

[15] Angi M , Kamath V , Yuvarani S , et al. The t(8;14)(q24.1;q32) and its variant translocations: a study of 34 cases. Hematol Oncol Stem Cell Ther. 2017;10(3):126‐134. 28390216 10.1016/j.hemonc.2017.03.002

